# Kenaf Fiber/Pet Yarn Reinforced Epoxy Hybrid Polymer Composites: Morphological, Tensile, and Flammability Properties

**DOI:** 10.3390/polym13091532

**Published:** 2021-05-10

**Authors:** M. J. Suriani, Hasliana Asyikin Zainudin, R. A. Ilyas, Michal Petrů, S. M. Sapuan, C. M. Ruzaidi, Rohani Mustapha

**Affiliations:** 1 Faculty of Ocean Engineering Technology and Informatics, Universiti Malaysia Terengganu, Kuala Nerus 21030, Terengganu, Malaysia; hasliana.zainudin@yahoo.com (H.A.Z.); ruzaidi@umt.edu.my (C.M.R.); rohani.m@umt.edu.my (R.M.); 2 Marine Materials Research Group, Faculty of Ocean Engineering Technology and Informatics, Universiti Malaysia Terengganu, Kuala Nerus 21030, Terengganu, Malaysia; 3 School of Chemical and Energy Engineering, Faculty of Engineering, Universiti Teknologi Malaysia, UTM, Johor Bahru 81310, Johor, Malaysia; 4 Centre for Advanced Composite Materials, Universiti Teknologi Malaysia, UTM, Johor Bahru 81310, Johor, Malaysia; 5 Faculty of Mechanical Engineering, Technical University of Liberec, Studentská 2, 461 17 Liberec, Czech Republic; michal.petru@tul.cz; 6 Laboratory of Biocomposite Technology, Institute of Tropical Forestry and Forest Products (INTROP), Universiti Putra Malaysia, UPM, Serdang 43400, Selangor, Malaysia; sapuan@upm.edu.my; 7 Advanced Engineering Materials and Composites Research Centre (AEMC), Department of Mechanical and Manufacturing Engineering, Faculty of Engineering, Universiti Putra Malaysia, UPM, Serdang 43400, Selangor, Malaysia

**Keywords:** kenaf composite, flammability, fire retardant, hybrid composite, tensile, morphology, pet yarn, epoxy

## Abstract

The application of natural fibers is rapidly growing in many sectors, such as construction, automobile, and furniture. Kenaf fiber (KF) is a natural fiber that is in demand owing to its eco-friendly and renewable nature. Nowadays, there are various new applications for kenaf, such as in absorbents and building materials. It also has commercial applications, such as in the automotive industry. Magnesium hydroxide (Mg(OH)_2_) is used as a fire retardant as it is low in cost and has good flame retardancy, while polyester yarn (PET) has high tensile strength. The aim of this study was to determine the horizontal burning rate, tensile strength, and surface morphology of kenaf fiber/PET yarn reinforced epoxy fire retardant composites. The composites were prepared by hybridized epoxy and Mg(OH)_2_ PET with different amounts of KF content (0%, 20%, 35%, and 50%) using the cold press method. The specimen with 35% KF (epoxy/PET/KF-35) displayed better flammability properties and had the lowest average burning rate of 14.55 mm/min, while epoxy/PET/KF-50 with 50% KF had the highest tensile strength of all the samples. This was due to fewer defects being detected on the surface morphology of epoxy/PET/KF-35 compared to the other samples, which influenced the mechanical properties of the composites.

## 1. Introduction

In the last few decades, advanced application of composite materials has gained a lot of interest from various sectors, especially transmission towers, defense, aircrafts, marine, and the automotive industry [1,2,3,4]. Industries have sought environmentally friendly materials for their products due to increasing global awareness about the environmental impact [5,6]. Natural fibers are renewable sources that have emerged as a new generation of reinforcement and supplement for polymers [7,8]. Growing industrial activities have prompted continuous demand for better materials that satisfy various requirements, such as higher strength, higher modulus, reduced cost, etc. These requirements often involve a combination of many properties that are difficult to attain. The use of a composite material whose constituents will synergize to solve the needs of the application is therefore necessary. Many researchers in the past have developed composites using natural fibers, such as sugar palm [9,10,11,12,13,14,15], oil palm [16], sugarcane [17,18,19], water hyacinth [20], kenaf [21,22,23], corn husk [24], bamboo [25], coir [26], sisal [27], cogon [28], and banana [29,30]. These natural fibers possess good reinforcing capability when properly combined with polymers [31].

Nowadays, hybrid composites are widely used in industrial and automobile applications. A hybrid composite can be defined as a combination of two or more different types of fibers in which one fiber balances the deficiency of another fiber [32,33,34,35]. They are composites with two or more reinforcing materials incorporated in a mixture and filling materials in a single matrix [36]. The properties of hybrid composite are influenced by the fiber content, length, and orientation. The selection of the fiber constituent for hybrid composites affects the hybridization and the requirement of the material being constructed. The selection of compatible fibers and fiber properties is therefore a critical aspect in designing hybrid composites [37]. This step is crucial in order to achieve hybrid composites with the best performance [38]. A previous study found that sisal and oil palm fibers were compatible combinations for hybrid composites due to the high tensile strength and toughness of the sisal and oil palm fibers, respectively [39]. In addition, there are some specific advantages of hybrid composites compared to conventional composites. For instance, hybrid composites can balance strength and stiffness, improve fatigue resistance, balance bending and membrane mechanical properties, increase fracture toughness, and improve impact resistance [40]. The advantages of hybrid composites could complement other fiber constituents. The hybridization of kenaf with polyester (PET) and polyoxymethylene (POM) composites improved the mechanical performance of the composites due to the high strength of PET fiber [41]. Ramesh et al. [42] studied the effect of hybridization on mechanical properties for sisal, jute natural fiber, and glass fiber woven mat. They found that a sisal/jute/glass composite enhanced the mechanical properties compared to a composite made of only sisal and jute fibers. Polymer composites can be hybridized with different combinations, such as cellulosic/cellulosic, synthetic/synthetic, and cellulosic/synthetic fibers [43]. As for cost reduction of fibers such as carbon and glass, an attempt was made by combining jute, a cost-effective and eco-friendly natural fiber, with fiber metal laminate (FML) [44]. The use of natural resources to make high-performance materials is increasing [45]. Fiber hybridization with synthetic fibers results in improvement of the properties of hybrid composites by increasing their strength [46,47]. Synthetic fibers have good potential for hybridization with natural fibers due to their low cost and high strength. PET fibers have good mechanical properties and hydrophobic characteristics, which means hybridization of kenaf fiber with PET yarn could improve the strength of the composite material.

Natural fibers contain hydrogen bonds and other linkages that give them strength and stiffness. In Western Europe, natural fibers are being used in the automotive industry and for packaging materials. Natural fibers are cellulosic materials, which have several fibrils that run all along the length of the fiber [48]. The demand for natural fiber composites has grown rapidly for various applications, such as automotive components, building materials, and the aerospace industry, due to their ecological and economic advantages compared to conventional composites [49]. The use of sisal, bamboo, sugar palm, kenaf, cotton, and jute from natural resources has helped engineers develop high-performance and environmentally safe engineering products.

One of the potential constituents of natural fiber reinforced plastic composites in Malaysia is kenaf fiber (KF). Research on kenaf has grown tremendously. Kenaf long fibers could be used for a wide variety of applications if the properties were found to be comparable to existing synthesis composites. Nowadays, there are various new applications for kenaf, such as in furniture, composite boards, automotive panels, insulation mats, geotextiles, packaging, absorbents, building materials, and paper products. Kenaf also has commercial applications, such as in the automotive industry [37,50,51]. Large global corporations, such as Toyota Motor Corporation, have led the world in the use of kenaf. Biocomposites can result in a 25% reduction of vehicle weight, consequently contributing to saving 39.45 trillion of crude oil [52]. Moreover, this material can be used for composite frames for electric vehicles as it will reduce energy consumption. Because kenaf is always available in long fiber form, the mechanical properties could be of use in many industrial applications, such as insulator seals. In addition, kenaf fibers have the advantage of being biodegradable, low density, nonabrasive during processing, and environmentally safe [53]. The attractive features of kenaf fibers are low cost, lightweight, renewability, biodegradability, and high specific mechanical properties. Kenaf has a bast fiber that contains 75% cellulose and 15% lignin and offers the advantage of being biodegradable and environmentally safe [54]. Kenaf, like all agro (lignocellulosic) fibers or cell wall polymers, is a three-dimensional polymeric composite primarily made up of cellulose (56–64%), hemicelluloses (29–35%), lignin (11–14%), and small amounts of extractives and ash.

When using natural fibers as reinforcement, the composite is prone to fire damage, and it is therefore essential to find additives that have low total emission of heat [55]. Therefore, it is really important to improve the flame retardancy of composite materials [49]. With natural fibers being increasingly used, the development of safe and environmentally friendly flame retardant polymer composites is very important. Previous studies have used magnesium hydroxide and zinc borate as flame retardants to enhance the mechanical and flammability properties of sisal/polypropylene (PP) composites. They showed that addition of flame retardant to sisal/PP composites increased the thermal stability of the composites and reduced burning. Just like other organic materials, polymers and natural fibers are very sensitive to flame. In order to minimize the fire risk and meet fire safety requirements, solutions to prevent the ignition of these materials have been developed. Thus, flame retardant composites were produced [56]. Flame retardants are chemical substances that are added to materials both to constrain and delay the spread of fire after ignition [57]. Magnesium hydroxide is low in cost and has good flame retardancy for use as a fire retardant. It is an environmentally friendly, inorganic flame retardant with low smoke and nontoxic characteristics [58]. Besides examining the addition of flame retardant material in natural fiber/polymer composites, there is also research being undertaken for treatment of natural fibers with fire retardant materials. The preparation and characterization of thermosetting and thermoplastic composite materials reinforced with the most commonly used fibers with and without treatment using different methods have been conducted in the past. Treatment using alkaline on the surface of the fiber changes the surface wettability, altering the mechanical and physical properties of the natural fibers. Previous studies have also shown that, as the fiber content increases in accordance with the mixture rule, the strength and modulus of longitudinal composites in tensile and flexural loading also increases [59].

Compared to other natural fibers, kenaf is eco-friendly and has a high growth rate. Therefore, in the present work, an investigation was carried out to study the horizontal burning rate, with magnesium hydroxide used to determine its capability as a fire retardant composite. Different percentages of kenaf fibers were added, with PET yarn and magnesium hydroxide used as controls. The objective was to determine the horizontal burning rate, tensile strength, and surface morphology of kenaf fiber/PET yarn reinforced epoxy fire retardant composites.

## 2. Materials and Methods

### 2.1. Materials

The main materials used in the study were kenaf fiber and PET yarn. Kenaf fiber was ordered from Innovative Pultrusion Sdn. Bhd., Negeri Sembilan. First, the kenaf fibers were combed to clean and untangle the strong bonds between individual fibers. Combed fibers exhibit stronger mechanical properties compared to uncombed ones [60]. Then, the kenaf fibers were cut to 22 cm pieces using scissors. PET yarn was bought from Wellward Sdn. Bhd., Johor, and were also cut into 22 cm pieces using scissors. The matrix used in this research was epoxy resin and hardener supplied by HR Team Enterprise, Kuala Terengganu, Terengganu. Magnesium hydroxide was ordered from HR Team Enterprise, Kuala Terengganu, Terengganu. Table 1 shows the properties of the epoxy resin and hardener. The densities of the materials used in this research are shown in Table 2.

### 2.2. Research Methodology

**Figure 1:**
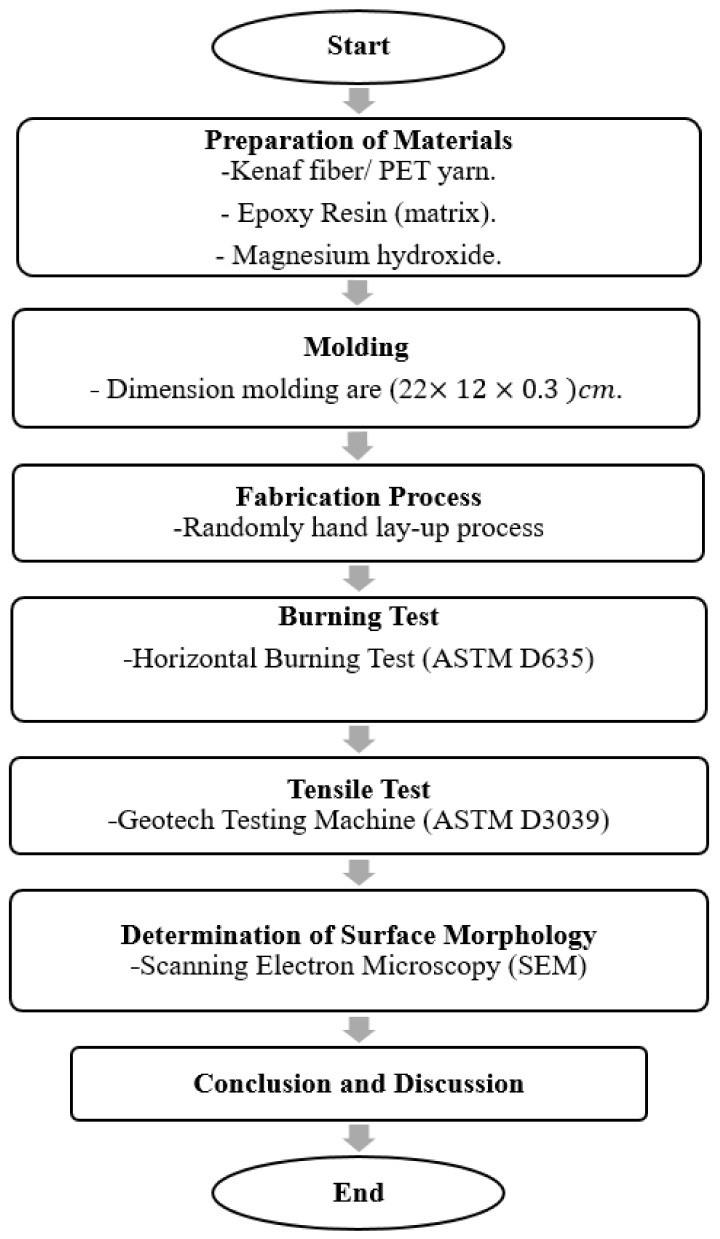
Research methodology.

Figure 1 shows the research methodology of the experiment. The experiment started with the preparation of materials, followed by fabrication of hybrid composites, and then tests carried out to determine burning, tensile, and surface morphology properties of the hybrid composites. The data obtained were analyzed and are critically discussed.

### 2.3. Preparation of Materials

The composite comprised a hybrid of kenaf fiber/PET yarn as reinforcement, while epoxy resin was used as the matrix and magnesium hydroxide as the fire retardant [61]. Epoxy with a density of 1.21 g/cm^3^ was used as a binding material to fabricate the composite specimen. The recommended mixing ratio for epoxy resin and hardener is 2:1. The volume of epoxy used depended on the different ratios of kenaf fiber/epoxy, which determined the weight of epoxy needed. In this study, magnesium hydroxide was used as the control and a high filled-type inorganic flame retardant, with 5% magnesium hydroxide mixed with the epoxy resin to enhance the flame retardancy of the composite material.

### 2.4. Fabrication Process

The composite samples were prepared using a hybrid of kenaf fibers/PET yarn with the epoxy resin as binder and magnesium hydroxide as fire retardant through a random hand lay-up technique. The composition of the samples of kenaf fiber reinforced epoxy fire retardant composites is shown in Table 3.

Before fabricating, kenaf fibers were combed and then cut into 22 cm pieces. After that, the fibers were weighed based on the density of the fiber to determine the exact portion of fiber that should be used in the fiber polymer composite samples to obtain the designated fiber volume content. One of the problems with kenaf fiber reinforced epoxy that needs to be addressed is uneven fiber distribution [62]. In addition, it is difficult to manually and visually separate kenaf fibers during manufacturing [63].

The fabricating process of the composites in this study was as follows. The molds were polished using grease oil (releasing agent) before starting fabrication. Epoxy resin was mixed with a ratio of 2:1 (resin: hardener by weight) according to the manufacturer’s specifications. The mixture was stirred slowly for 10 minutes to ensure that both epoxy and hardener were uniformly mixed. Then, magnesium hydroxide was poured into the mixture of epoxy and hardener. The mixture of magnesium hydroxide was then stirred slowly for 5–10 min to ensure the mixtures were uniformly mixed.

**Figure 2:**
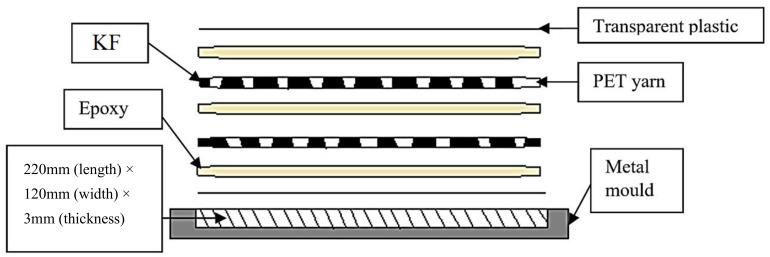
Arrangement of kenaf fiber and PET yarn with epoxy in the metal mold.

A transparent plastic sheet was placed on the surface of the mold. Then, the mixture of magnesium hydroxide with epoxy and hardener was poured slowly into the mold. Next, the fibers and PET yarn were completely immersed in resin by portion and then distributed randomly in the mold. The composite was covered by a transparent plastic sheet to avoid formation of air bubbles inside the mold. The plastic cover was used to prevent the fibers from attaching to the steel plate during curing. The composite was pressed using a flat plate to remove the remaining bubbles inside the mold. The mold was then compressed by another steel plate on top of the transparent plastic sheet. Demoulding of specimens was carried out after 24 h of fabrication. Figure 2 depicts the arrangement of KF and PET yarn with epoxy in the metal mold. Finally, the mold was cleaned to be used for the next specimen.

**Figure 3:**
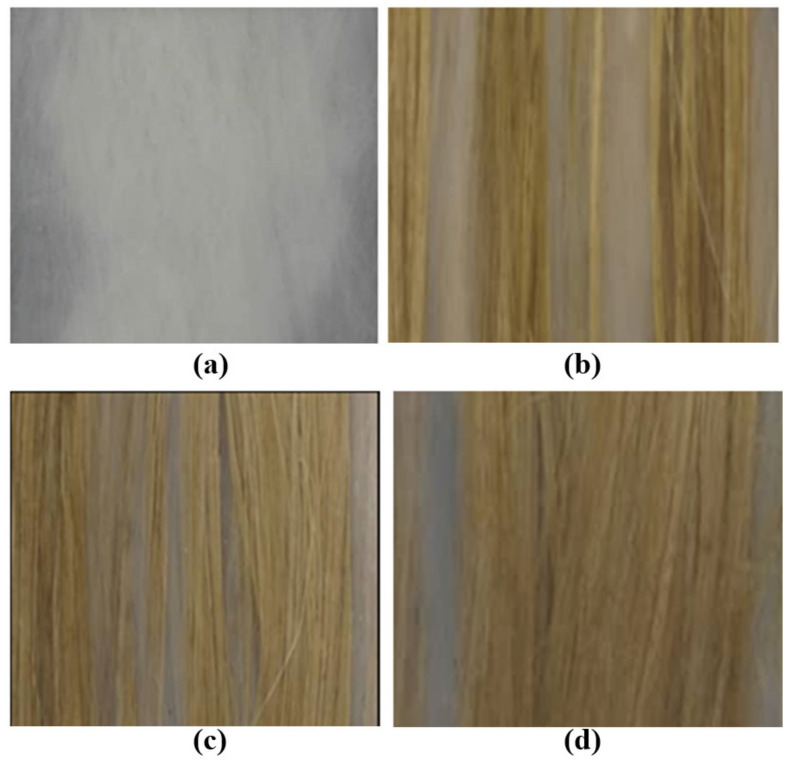
Observation of sample: (**a**) epoxy/Mg/PET, (**b**) epoxy/PET/KF-20, (**c**) epoxy/PET/KF-35, and (**d**) epoxy/PET/KF-50.

For each composition, the fiber and its binder were calculated to determine the weight of the fiber and epoxy needed to fill the mold. After fabrication of the composite was complete, the specimen of each composition was cut into nine pieces of 1.3 cm width for use in the horizontal burning test and eight pieces of 1.5 cm width for use in the tensile test. The images of these composites were also captured after the tensile test. Table 4 shows the results for all samples. Figure 3 presents the samples of each composition that were completely cured.

### 2.5. Testing and Experiment

#### 2.5.1. Horizontal Burning Test

The burning test was conducted to determine the burning rate of the samples. The samples were set up according to ASTM standard D635. For these points, all specimens were cut to exact length with dimension of 12.5 cm length × 1.3 cm width × 0.3 cm thickness. The test was carried out in triplicate. The test samples were supported horizontally at one end. Next, the free end was exposed to a specified gas flame for 30 s. The time and extent of burning were measured using a stopwatch, and it was noted if the specimen did not burn. An average burning rate was noted for the material if it was burned to the 100 mm mark from the ignited end. The formula below was used to determine the average burning rate of each sample:

(1) $$\mathrm{Average}\;\mathrm{burning}\;\mathrm{rate}\;\left(\mathrm{mm}/\min\right)=\frac{\mathrm{Damaged}\;\mathrm{length},\;L\;\left(\mathrm{mm}\right)\;}{\mathrm{Time},\;t\;\left(\min\right)}$$

#### 2.5.2. Tensile Test

The fiber tensile test was used to determine the strength of the composite materials. The samples were set up according to ASTM standard D3039M. First, all of the samples were cut into the same size of 20 cm height × 1.5 cm width × 0.3 cm thickness. Then, four aluminum plates with a dimension of 25 mm × 15 mm × 1 mm were attached to the two sides of both ends of the samples using the epoxy resin. The test was carried out in five replications using the Universal Testing Machine (INSTRON 5556) (Norwood, MA, USA) with a 5 kN load cell; the crosshead speed was maintained at 2 mm/min.

#### 2.5.3. Scanning Electron Microscopy

**Figure 4:**
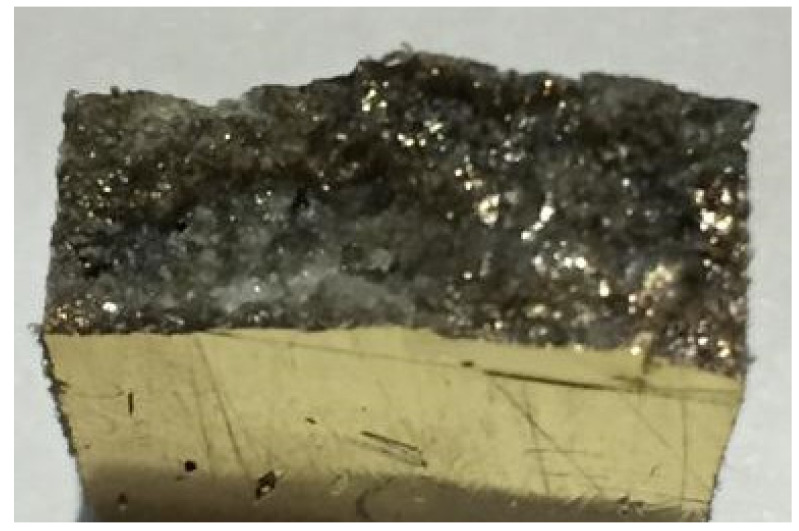
Cross section of sample after being coated with gold.

SEM is capable of performing analyses of selected point locations on a sample, which is especially useful for qualitatively or semiquantitatively determining chemical composition (using energy-dispersive (EDS)), crystalline structure, and crystal orientations (using electron backscatter diffraction (EBSD)). The design and function of the SEM are very similar to EPMA, and considerable overlap in capabilities exists between the two instruments. From the tensile test fracture samples, the samples at the edge of the fracture side were cut using a jigsaw machine. Then, the samples were coated with gold using a vacuum sputter coater to make them conductive prior to SEM observation. The cross section of samples was coated with gold as shown in Figure 4. The samples underwent observation to detect defects on the surface using SEM.

## 3. Results and Discussion

### 3.1. Flammability Properties

**Figure 5:**
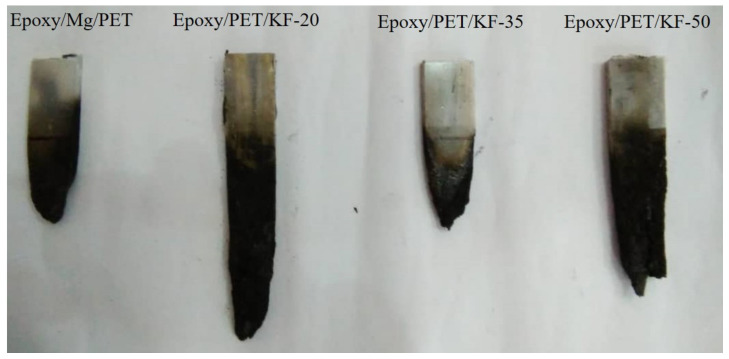
Samples of each composition after the burning test.

**Figure 6:**
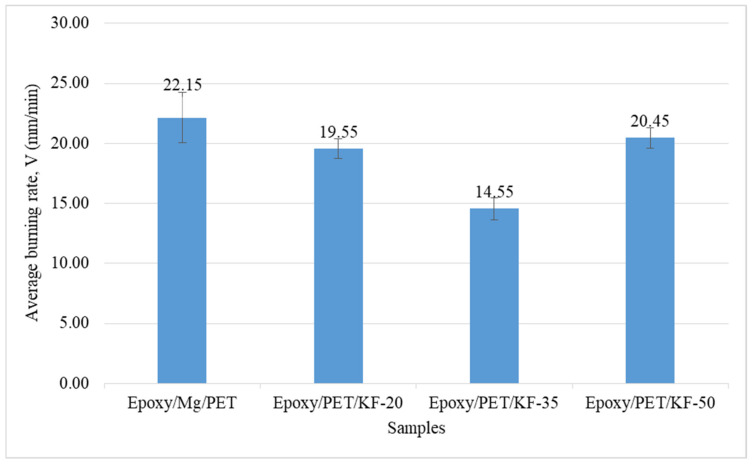
Graph of horizontal burning test for each composition.

From the results of horizontal burning test for every sample, the average horizontal burning rate was determined. Figure 5 shows samples of each composition after the burning test. The average burning rate for each sample was converted to a graph so that the trend of this average can be clearly seen (Figure 6).

From Figure 6, it can be clearly seen that the epoxy/Mg/PET with 0% KF showed the highest average burning rate of 22.151 mm/min compared to the other samples. Epoxy/Mg/PET ignited earlier, released more heat overall, and took shorter average time to reach its burning point. The ignition time is related to the fiber volume fraction of resin exposed to the flame on the composite surface [64]. Polymeric matrices have poor flammability behavior. The polymer matrix depends on the reinforcements and fillers, and it has no prominent role in improving the flame resistance of composites. The PET yarn was melted and moved away from the flame and burned with smoky flame during the burning test.

The KF content for epoxy/PET/KF-20, epoxy/PET/KF-35, and epoxy/PET/KF-50 was 20%, 35%, and 50%, respectively. The average burning rate of epoxy/PET/KF-35 was the lowest at 14.553 mm/min compared to the other samples. As a result, the composite with 35% KF and 5% magnesium hydroxide showed better result due to the compatible combination of fiber and fire retardant compared to 20% and 50% KF. The fiber volume content in epoxy/PET/KF-35 was the best compared to epoxy/PET/KF-20 and epoxy/PET/KF-50 samples because of the lower burning rate and higher fire retardant effectiveness. When the fiber content was increased above 20%, the characteristics of the composite became more similar to lignocellulosic materials [65]. A recent study showed that the presence of kenaf fiber increased the smoke density rating due to the characteristics of kenaf fiber that promote smoke production through char formation when exposed to flame [66].

The flame took a long time to propagate along epoxy/PET/KF-35 and also produced char at the same time. The production of char was the reaction of Mg in front of the flame. Charring is known as a chemical process of incomplete combustion of certain solids when subjected to high heat. By the action of heat, charring removes hydrogen and oxygen, so only carbon remains in the char. In addition, as acid and halogen-free flame retardant, Mg releases the water of hydration when it decomposes endothermically, which helps the flame retarding action [67]. The energy absorption mechanism then occurs because the magnesium compound undergoes a highly endothermic decomposition reaction that slows the heating rate of most materials in fire [68]. Epoxy/PET/KF-50 showed the highest average burning rate among the samples that contained the natural fiber. This might be due to possible environmental conditions that promote increased burning rate [66].

### 3.2. Tensile Properties

**Figure 7:**
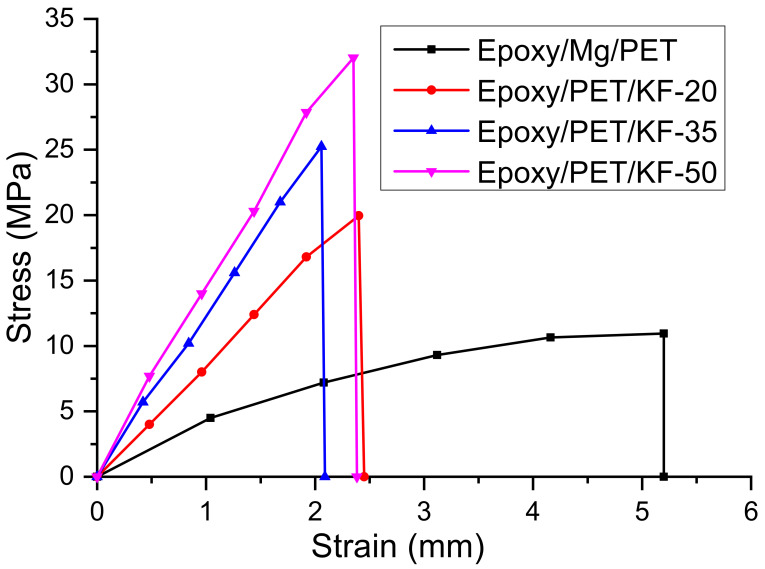
The stress–strain diagram of hybrid kenaf fiber/PET yarn reinforced epoxy fire retardant composites.

The ultimate tensile strength, elongation break, and elastic modulus were determined from the stress–strain curve of hybrid kenaf fiber/PET yarn reinforced epoxy fire retardant composite after the test. Statistical analysis was conducted based on the data of five specimens for each composite sample. Figure 7 shows the stress–strain curve of hybrid kenaf fiber/PET yarn reinforced epoxy fire retardant composites with different fiber volume contents (epoxy/Mg/PET, epoxy/PET/KF-20, epoxy/PET/KF-35, and epoxy/PET/KF-50), which was linear and followed the Hooke’s law. It can be seen that epoxy/PET/KF-50 had better tensile properties compared to the other samples. Its ultimate tensile stress was 32.02 MPa, which was higher than the those of epoxy/Mg/PET, epoxy/PET/KF-20, and epoxy/PET/KF-35, which were 10.87, 19.96, and 25.24 MPa, respectively.

**Figure 8:**
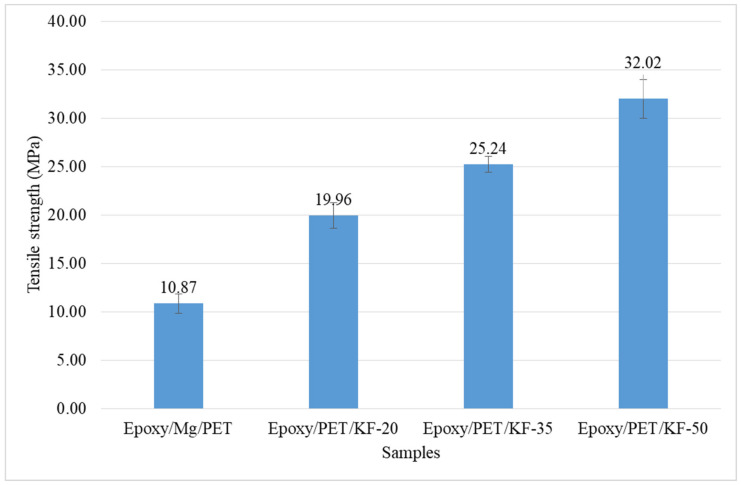
Effect of kenaf fiber loading on the tensile strength of epoxy/Mg/PET, epoxy/PET/KF-20, epoxy/PET/KF-35, and epoxy/PET/KF-50.

The tensile strength of hybrid kenaf fiber/PET yarn increased with the increase of fiber volume content, as shown in Figure 8. The tensile strength of the composites is tabulated in Table 5. The combination of kenaf fiber and PET yarn resulted in an enhancement of tensile properties of the composites. The ultimate strain of the epoxy/Mg/PET composite was the lowest compared to the other samples. The tensile strength is greatly dependent on effective and uniform stress distribution [69,70,71]. The increased strength of the hybrid composite was primarily due to the high strength of the hemp fiber rather than the low strength of the PET fiber [72].

According to the study by Mobedi et al. [73], the addition of magnesium hydroxide has a negative impact on the tensile properties, with magnesium hydroxide found to be a cursor for controlling the polymer degradation rate and accelerating the rate of degradation of composites. A previous study revealed that the deterioration of strength was insignificant with addition of 15% and 20% magnesium hydroxide [74]. However, in this study, the epoxy/Mg/PET composite had the lowest tensile strength. This might be due to the defects that occurred in the composites during fabrication. It might have also been caused by the addition of only 5% (control) magnesium hydroxide, which did not have much influence on the tensile strength compared to the increase in percentage of KF.

### 3.3. Surface Morphology

The surface morphology of all the specimens after the tensile test was studied by capturing images of the fractured area. From this analysis, it was concluded that several types of defects occurred on the specimens. Defects typically take place during the production of composites and sometimes by manual construction known as “lay-up”. Variations in the amount of defects in composites during manufacturing will increase the likelihood of composite failure.

**Figure 9:**
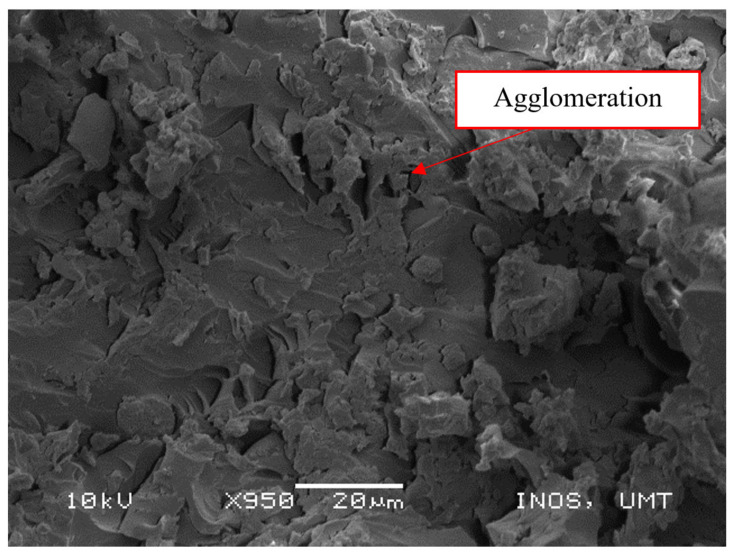
Agglomeration of Mg(OH)_2_ in the epoxy/Mg/PET sample.

Two problems are critical to the material response: defects (built-in at manufacture) and damage (changes due to use). The effects of damage have been extensively covered in the open literature. Heslehurst and Scott [75] found that the “level of structural degradation in engineering properties varied” with defect severity, defect location and orientation, frequency of defect occurrence, component load path criticality and stress state, and defect idealization. As shown in Figure 9, the presence of agglomeration could be seen in the SEM micrograph of the epoxy/Mg/PET sample. It is known that Mg(OH)_2_ has a great tendency to form agglomerates. In fact, agglomeration is a well-known phenomenon, and its probability increases with decreasing particle size. These agglomerated particles could be stress concentrator points and could affect the final performance of the composites. This might result from the defects that occur in the composites during fabrication. Moreover, it is possible to identify the needled structure of Mg(OH)_2_ particles in images of higher magnification, e.g., 950×. This behavior might be an indication of poor interaction between the matrix and Mg(OH)_2_ [76].

**Figure 10:**
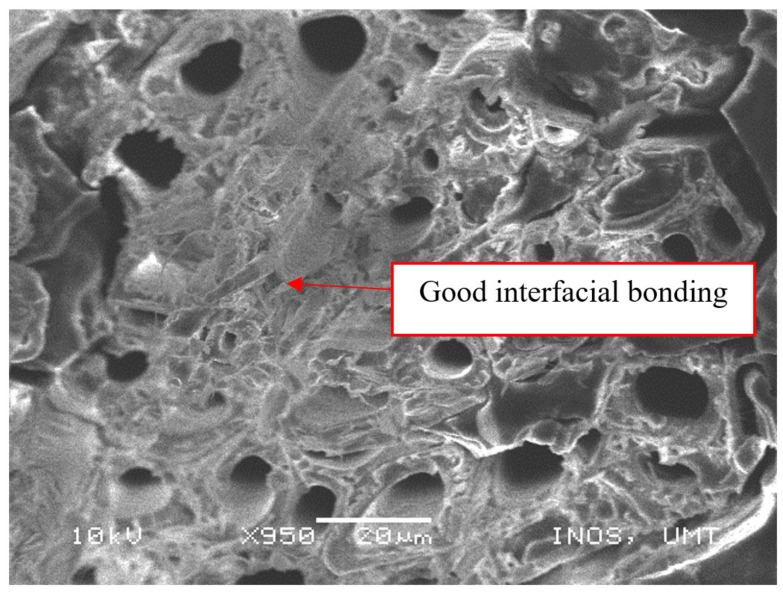
Good interfacial bonding in the epoxy/PET/KF-20 sample.

**Figure 11:**
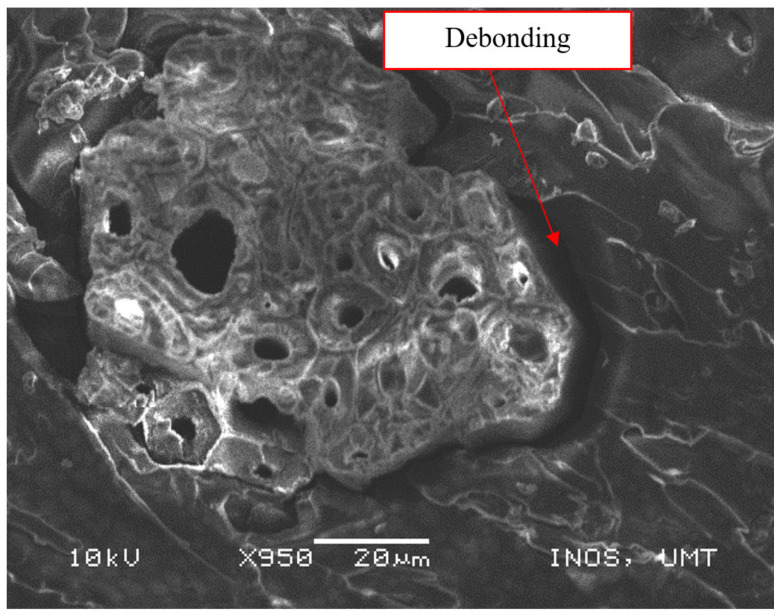
Debonding of fiber and polymer in the epoxy/PET/KF-35 sample.

As shown in Figure 10, there was good interfacial bonding of matrix and fiber in the epoxy/PET/KF-20 sample. The effective stress transfer between the fiber and the matrix was due to good interfacial bonding and was further promoted by maximizing the fiber strength [43]. The small particles of magnesium hydroxide possessed good dispersity and compatibility in the matrix, which provided a strengthening and toughening effect. Therefore, the tensile strength and elongation at break were substantially increased. Figure 11 presents the SEM micrograph of the tensile-fractured surface for debonding of the fiber within the epoxy/PET/KF-35 sample. The poor compactness of the composite caused weaker bonding strength between the matrix and the fiber.

**Figure 12:**
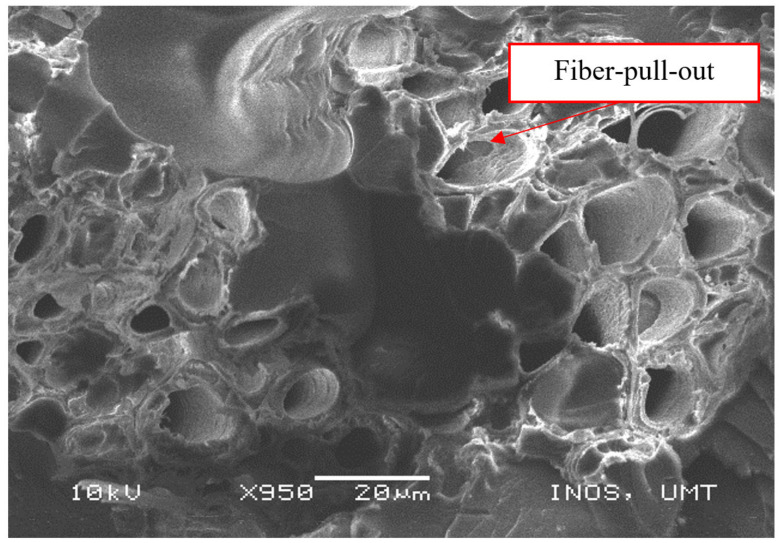
Fiber pull-outs in the epoxy/PET/KF-50 sample.

Figure 12 shows the tensile damage that occurred on the composite, which consisted of fiber pull-outs and ensured enhanced interfacial adhesion in the epoxy/PET/KF-50 sample. As reported by Sanadi et al. [77], the primary disadvantage of natural fibers is the poor interfacial adhesion due to the hydrophilic character of cellulose, which is commonly mismatched with the hydrophobic matrix material [78,79]. Therefore, this phenomenon leads to poor fiber dispersion and fiber–matrix interfacial adhesion. This might also be due to the high fiber content, with the quantity of matrix not enough to cover the fiber [80,81,82].

## 4. Conclusions

In order to reduce cost and provide a pollution-free environment, the flammability and mechanical properties of hybrid kenaf fiber/PET yarn reinforced epoxy fire retardant composites was analyzed. The horizontal burning test showed that epoxy/Mg/PET with 0% KF had the highest average burning rate of 22.151 mm/min compared to epoxy/PET/KF-20, epoxy/PET/KF-35, and epoxy/PET/KF-50, which contained 20, 35, and 50% KF, respectively. The average burning rate of epoxy/PET/KF-35 was the lowest at 14.553 mm/min compared to the other samples. This suggests that epoxy/PET/KF-35 would be better for application as a fire retardant composite because of the optimum composition. Epoxy/PET/KF-50 demonstrated the highest tensile strength of 32.022 MPa compared to the other samples. The tensile strengths of epoxy/Mg/PET, epoxy/PET/KF-20, and epoxy/PET/KF-35 were 10.867, 19.956, and 25.244 MPa, respectively. The tensile strength of the hybrid kenaf fiber/PET yarn increased with the increase of fiber volume content. The combination of kenaf fiber and PET yarn enhanced the tensile properties of the composites. In addition, too many defects result in poor mechanical properties of composites. Epoxy/PET/KF-50 showed the fewest defects. In general, the surface morphology of the hybrid kenaf fiber/PET yarn possessed defects such as fiber pull-out and debonding.

## Figures and Tables

**Table 1:** Properties of epoxy resin and hardener.

Property	Epoxy Resin	Hardener
Form	Liquid	Liquid
Density (g/cm^3^)	1.21	1.03
Curing time (hours)	24	24
Ratio	2	1

**Table 2:** Densities of materials used.

Material	Density (g/cm^3^)
Kenaf	0.37
PET yarn	1.38
Epoxy	1.21
Magnesium hydroxide (Mg(OH_2_)) powder	1.20

**Table 3:** Composition of samples.

Sample Designation	Percentage (%)			
	KF	PET Yarn	Mg(OH)_2_	Epoxy + Hardener
Epoxy/Mg/PET	0	5	5	90
Epoxy/PET/KF-20	20	5	5	70
Epoxy/PET/KF-35	35	5	5	55
Epoxy/PET/KF-50	50	5	5	40

**Table 4:** Results of the calculation for all samples.

Descriptions	Samples			
	Epoxy/Mg/PET	Epoxy/PET/KF-20	Epoxy/PET/KF-35	Epoxy/PET/KF-50
Volume of specimen	79.2 cm^3^	79.2 cm^3^	79.2 cm^3^	79.2 cm^3^
Volume of fiber	0 cm^3^	15.84 cm^3^	27.72 cm^3^	39.6 cm^3^
Weight of fiber, W_f_	0 g	18.37 g	32.16 g	45.94 g
Volume of PET yarn	3.96 cm^3^	3.96 cm^3^	3.96 cm^3^	3.96 cm^3^
Weight of PET yarn	5.46 g	5.46 g	5.46 g	5.46 g
Volume of fire retardant	3.96 cm^3^	3.96 cm^3^	3.96 cm^3^	3.96 cm^3^
Weight of fire retardant	9.28 g	9.28 g	9.28 g	9.28 g
Volume of matrix	71.28 cm^3^	55.44 cm^3^	43.56 cm^3^	31.68 cm^3^
Weight of matrix, W_m_	85.54 g	66.58 g	52.27 g	38.02 g
Weight of epoxy	57.02 g	44.38 g	34.85 g	25.34 g
Weight of hardener	28.51 g	22.19 g	17.42 g	12.67 g

**Table 5:** Mechanical properties of hybrid kenaf fiber/PET yarn for all samples.

Samples Designation	Tensile Strength (MPa)	Elastic Modulus (MPa)
Epoxy/Mg/PET	10.87 ± 1.92	326.16 ± 20.13
Epoxy/PET/KF-20	19.96 ± 2.49	598.66 ± 15.12
Epoxy/PET/KF-35	25.24 ± 1.56	757.49 ± 25.95
Epoxy/PET/KF-50	32.02 ± 3.91	960.80 ± 30.87

## Data Availability

The data presented in this study are available on request from the corresponding author.
